# Among the Colleges

**Published:** 1898-10

**Authors:** 


### AMONG THE COLLEGES.

Every Western veterinary college reports brighter prospects and promised increased attendance of students.

Kansas City Veterinary College opened Wednesday, September 13, 1898, with a much larger class than for several years.

The McKillip Veterinary College of Chicago since the announcement was sent forth has provided for a very complete course of meat-inspection and preparation for government positions in the Bureau of Animal Industry. This course will be conducted by S. G. Burkholder, M D., V.S., connected with the Federal meat-inspection service at Chicago, and will embrace comparative anatomy, comparative physiology, comparative histology, gross and minute pathology, bacteriology, helminthology, antemortem inspection, postmortem inspection, and department regulations. Practical demonstrations at the Union Stock Yards.

The Western Veterinary College, of Kansas City, will open about October 1st, and expects a class of twenty students. The lectures will be given in an office room near Twelfth and Cherry Streets, rented for the purpose. Dissections will be conducted in conjunction with facilities afforded for clinical study in connection with the practice of Dr. J. H. Wattles, the promoter of the new institution.

The Ontario Veterinary College reopens its sessions of 1898–99 on Wednesday, October 12th. The catalogue for the ensuing year has just been issued, to which are added the names of the graduates of the class of 1898. The school has the largest list of graduates of any institution in North America.

